# Reproductive Isolation among Sympatric Molecular Forms of *An. gambiae* from Inland Areas of South-Eastern Senegal

**DOI:** 10.1371/journal.pone.0104622

**Published:** 2014-08-06

**Authors:** El Hadji Amadou Niang, Lassana Konaté, Mawlouth Diallo, Ousmane Faye, Ibrahima Dia

**Affiliations:** 1 Unité d’Entomologie Médicale, Institut Pasteur de Dakar, Dakar, Sénégal; 2 Laboratoire d’Ecologie Vectorielle et Parasitaire, Université Cheikh Anta Diop de Dakar, Dakar, Sénégal; National Institute for Communicable Diseases/NHLS, South Africa

## Abstract

The *Anopheles gambiae* species complex includes at least seven morphologically indistinguishable species, one of which, *Anopheles gambiae sensu stricto,* is the primary mosquito vector responsible for the transmission of malaria across sub-Saharan Africa. Sympatric ecological diversification of *An. gambiae* s.s. is in progress within this complex, leading to the emergence of at least two incipient species (the M and S molecular forms now recognized as good species and named *An. coluzzii* and *An. gambiae* respectively) that show heterogeneous levels of divergence in most parts of Africa. However, this process seems to have broken down in coastal areas of West Africa at the extreme edge of the distribution. We undertook a longitudinal study to describe *An. gambiae* s.s. populations collected from two inland transects with different ecological characteristics in south-eastern Senegal. Analysis of samples collected from 20 sites across these two transects showed the M and S molecular forms coexisted at almost all sampled sites. Overall, similar hybridization rates (2.16% and 1.86%) were recorded in the two transects; sites with relatively high frequencies of M/S hybrids (up to 7%) were clustered toward the north-western part of both transects, often near urban settings. Estimated inbreeding indices for this putative speciation event varied spatially (range: 0.52–1), with hybridization rates being generally lower than expected under panmictic conditions. Such observations suggest substantial reproductive isolation between the M and S molecular forms, and further support the ongoing process of speciation in these inland areas. According to a recent reclassification of the *An. gambiae* complex, the M and S molecular forms from this zone correspond to *An. coluzzii* and *An. gambiae*, respectively. There is considerable evidence that these molecular forms differ in their behavioural and ecological characteristics. Detailed study of these characteristics will allow the development and implementation of better insect control strategies for combating malaria.

## Introduction

Members of the *Anopheles gambiae* complex are the main vectors of malaria in most parts of Africa. This complex is made up of at least seven closely related species that cannot be distinguished using classic taxonomic methods [1]. Across their range, these species show different ecological and behavioural characteristics and include two of the most efficient human malaria vectors worldwide: *An. arabiensis* and *An. gambiae* sensu stricto that have the widest distribution of species in the complex [2].

Earlier studies addressing gene flow within the complex showed that *An. gambiae* s.s. is divided into reproductively isolated sub-populations. Five chromosomal forms were initially identified (Forest, Savanna, Bamako, Mopti, and Bissau), based on their patterns of chromosome 2 inversions [1], [3]. Studies to determine the taxonomic status of these chromosomal variants revealed that *An. gambiae* s.s. contains two molecular forms, M and S, which are recognizable by differences in their rDNA sequences [4], [5]. The S form is distributed widely throughout the *An. gambiae* species range, whereas the M form is restricted to western parts of Africa, where it is common. Hybridization between them is rare in most areas of sympatry [6]; extensive studies conducted throughout areas of sympatric distribution showed variable but restricted gene flow between the M and S forms [2], [6], [7]. This has resulted in the two forms being recognized as good species named *An. coluzzii* and *An. gambiae* respectively [8]. However, studies in coastal areas at the western extreme of the geographical range found a higher than expected hybridization rate. In areas surrounding The Gambia, high levels of M/S hybrids were reported at sites near the west coast (3% at Dielmo, Senegal [9]; 7% at Njabakunda, The Gambia [10]; and 24% at Antula, Guinea-Bissau [11]). More recently, Nwakanma et al. [12] reported high frequencies (5–42%) of M/S hybrid forms at 12 sites in the four contiguous countries of The Gambia, Senegal, Guinea-Bissau and Republic of Guinea. However, in other coastal areas of Africa the observed frequency of M/S hybrids did not exceed 0.2% [7], [13] and so it is unknown whether this phenomenon is specific to the westernmost areas of West Africa, including Senegambia. We undertook a longitudinal study within two transects under differential insecticide pressure in inland areas in south-eastern Senegal to (i) study the distribution patterns of the M and S molecular forms, (ii) estimate the frequencies of M/S hybrids in these sites, and (iii) use our observations to determine the extent of ongoing speciation in these inland areas.

## Materials and Methods

### Ethics statement

No specific permission was required for work in each of the selected villages. After explaining the purpose of this study, verbal consent was obtained from the village chief as well as from heads of households. The study did not involve endangered or protected species.

### Study area

**Figure 1:**
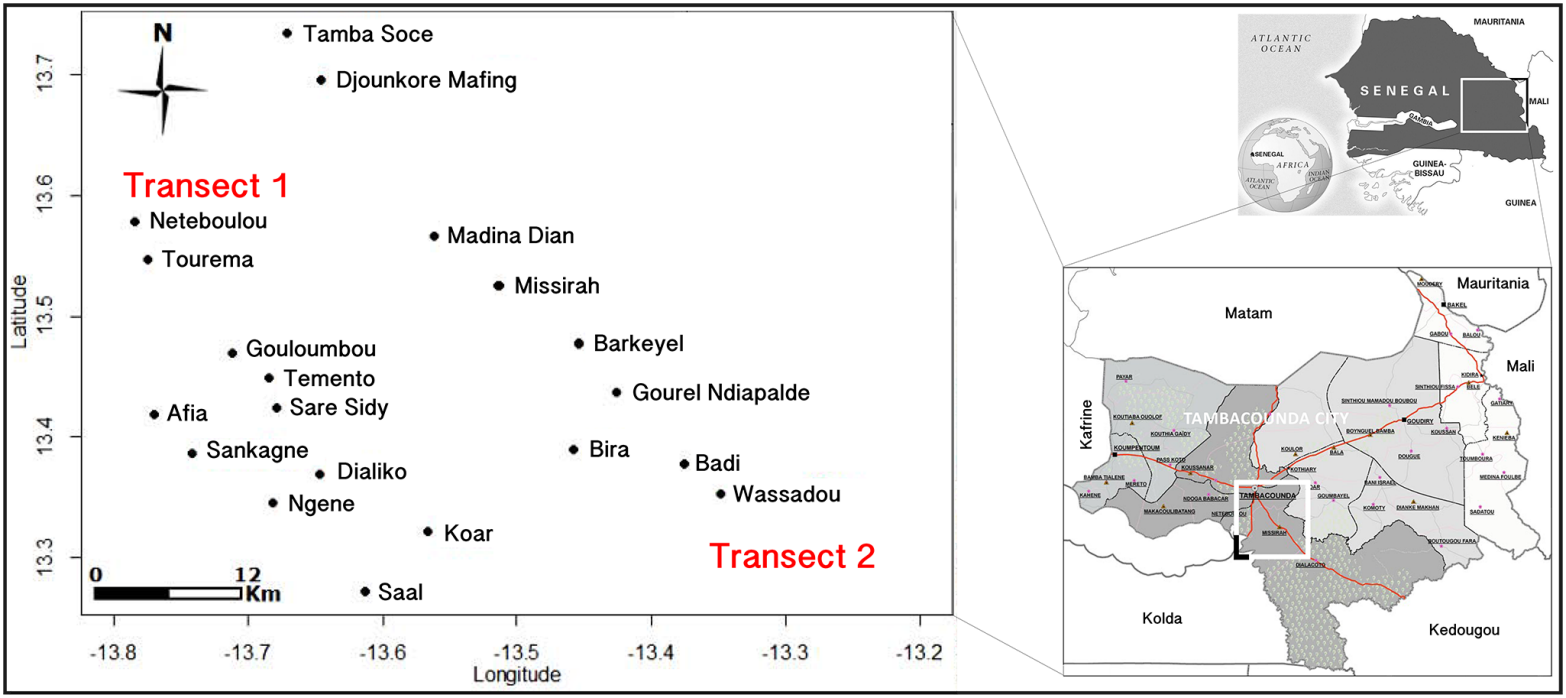
Localisation of the study sites.

The study was conducted in the Tambacounda region of south-eastern Senegal (Fig. 1). This region is characterised by savannah with trees and shrubs and by cultivated landscapes. It is divided by three main rivers (Senegal, Faleme and Gambia) and their numerous tributaries. The rainy season lasts from June–October, with a peak in August–September, and an annual rainfall of 500 mm. The estimated human population in 2009 was over 630, 000 inhabitants, dominated by Fulani and Manding.

Study sites were selected based upon the distribution of *An. gambiae* molecular forms across the area; selection focused on areas of sympatry determined in previous studies [6], [10]. Two distinct transects were identified based on their proximity to a river with wetlands and the type of agriculture practiced, particularly with respect to the use of pesticides for crop protection. The first transect (T1) was situated along the Gambia River in an area cultivating rice and cash crops of banana with a high usage of pesticide. Eleven villages were selected in this transect. All were within a rural freshwater flood plain in the vicinity of the Gouloumbou River and were surrounded by rice fields and banana plantations with moderate to high insecticide pressure. The second transect (T2), located along National Road 7, was selected for comparison with T1. It was in a less humid arid area with little or no application of pesticides. Nine villages were selected within this transect.

Study villages were selected within each transect using the Geographic Information System (GIS) software package, Arcview 3.2. The maximum and minimum geographical distances between two adjacent localities were 5 and 10 km, respectively, determined by visualization on a map with a buffer zone of 10 km set about a given location. The existence of previous entomological data and the accessibility of the study sites in all seasons were taken into account when selecting sites. All the selected villages, their GPS coordinates and their main characteristics are presented in Table 1 and File S1.

**Table 1:** Main characteristics of the study sites.

Sites ID	Transects/Sampling sites	Latitude N	Longitude W	Area type	Main human activities
	Transect 1				
1	Neteboulou	13°34	13°47	Semi-urban	Agro pastoral
2	Tourema	13°32	13°46	Rural	Agro pastoral
3	Gouloumbou	13°27	13°42	Semi-urban	Agro pastoral *&* trade
4	Afia	13°25	13°46	Semi-urban	Cash crop (Banana)
5	Temento Malede	13°26	13°41	Rural	Agro pastoral
6	Sare Sidy	13°25	13°40	Rural	Agro pastoral
7	Sankagne	13°23	13°44	Semi-urban	Cash crop (Banana)
8	Dialiko	13°21	13°38	Rural	Agro pastoral
9	Nguene	13°21	13°40	Rural	Cash crop (Banana)
10	Koar	13°20	13°37	Rural	Cash crop (Banana)
11	Saal	13°16	13°36	Rural	Cash crop (Banana)
	Transect 2				
12	Tamba Soce	13°46	13°40	Semi-urban	Agro pastoral
13	Djounkore Mafing	13°41	13°38	Rural	Agro pastoral
14	Madina Dian	13°33	13°33	Rural	Agro pastoral *&* trade
15	Missirah	13°31	13°30	Urban	Agro pastoral *&* trade
16	Barkeyel	13°28	13°27	Rural	Agro pastoral
17	Gourel Ndiapalde	13°26	13°25	Rural	Agro pastoral *&* trade
18	Bira	13°24	13°28	Rural	Agro pastoral
19	Badi	13°22	13°22	Rural	Agro pastoral *&* trade
20	Wassadou	13°21	13°20	Rural	Agro pastoral *&* trade

### Mosquito sampling and field processing

Ten randomly selected sleeping rooms in each of the 20 villages selected for study were visited monthly between July and December 2010. Mosquitoes resting indoors during the daytime were collected using pyrethrum sprays. Upon collection, mosquitoes were sorted, counted and identified morphologically using the keys of Gillies & De Meillon [14]. All the mosquito samples were stored individually after collection in numbered vials containing desiccant until laboratory processing.

### Laboratory processing

For molecular identification, genomic DNA was extracted from the wings or legs of individual mosquitoes as described by Collins et al. [15]. For each collection of mosquitoes randomly sampled from each village, 30–100% of females belonging to the *An. gambiae* complex were identified to the level of species and molecular form, using the molecular methods of Fanello et al. [5] and Favia et al. [16].

### Data analysis

The proportions of each species and molecular form were estimated and their means compared between study sites and transects by ANOVA. Prior to comparison, the constancy of variance (homoscedasticity) was checked using Bartlett and Fligner–Killeen tests. The frequencies of M/S hybrids were compared with Hardy-Weinberg expectations using the exact test procedures implemented in GENEPOP software (version 3.4) [17]. Moran’s *I* statistic [18] was used to determine spatial autocorrelation by measuring the correlation among neighbouring sites, to find the patterns and the levels of spatial clustering (i.e., clustered, dispersed, or random). The first step in the analysis is to construct a spatial weight matrix that contains information about the neighbourhood structure for each site. Adjacency is defined as immediately neighbouring sites, inclusive of the site itself. Non-neighbouring sites are given a weight of zero. The sites with high and low clustering were identified using the Getis-Ord Gi* statistic ([19] and File S2). Statistically significant (at a level of 0.05) clusters of sites with high M/S frequencies were identified with Z scores >1.96. Clustered sites with low M/S frequencies were identified with Z scores <–1.96.

The Moran scatterplot was used to classify local clustering further into four classes by comparing the frequency of M/S hybrids at each sampling site with that at neighbouring sites. All analyses were performed using R software (version 3.0.2).

## Results

### Mosquito collection

#### Species distribution

**Figure 2:**
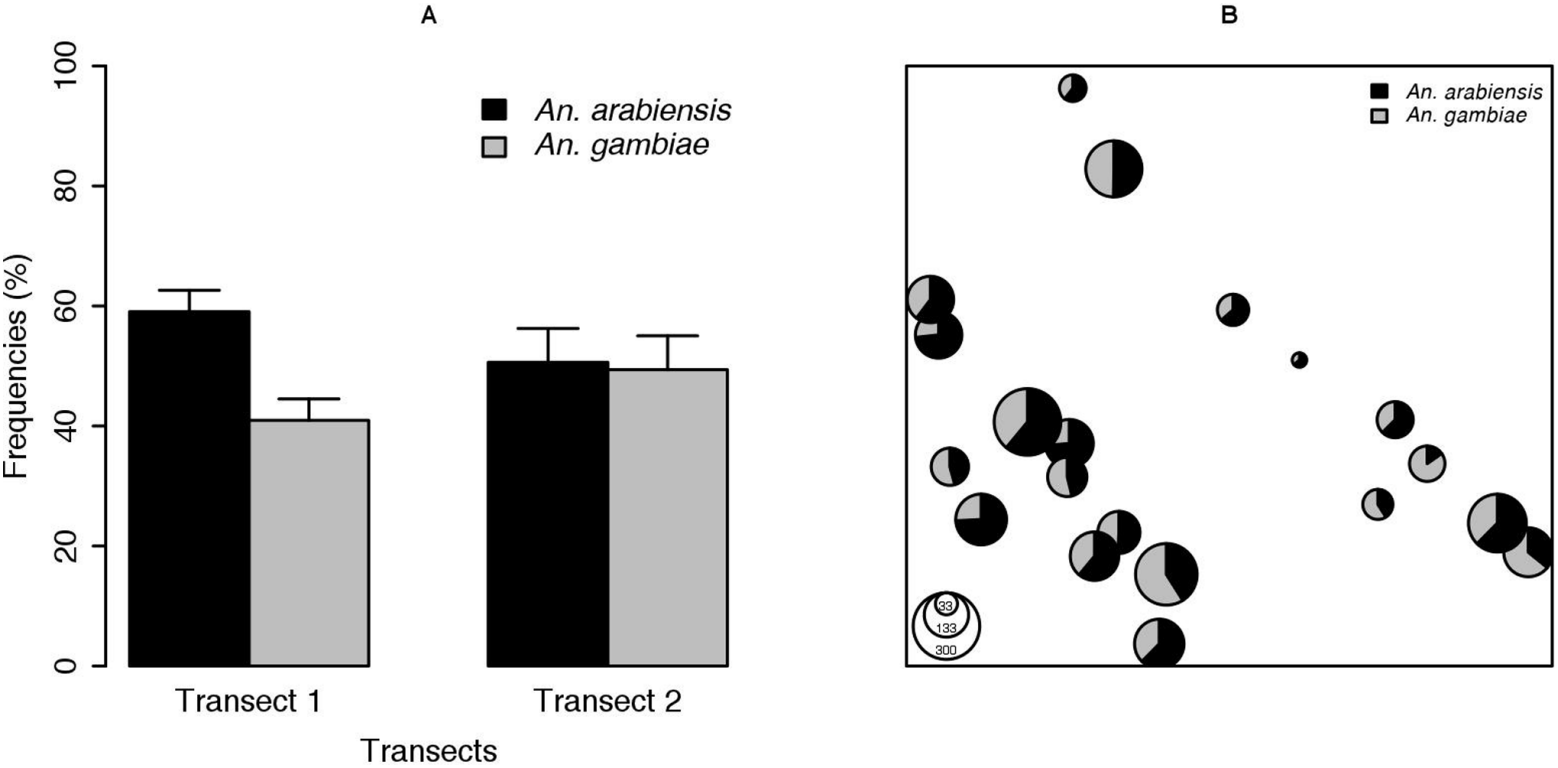
Variations of the mean frequencies of *An*. *arabiensis* and *An*. *gambiae*. A: between transects, bars represent standard errors. B: within villages in each transect (Transect 1 = T1, Transect 2 = T2).

Two species, *An. gambiae* s.s. and *An. arabiensis*, were identified among the 2,783 mosquitoes of the *An. gambiae* complex collected across the 20 sites (Table 1). They represented 44% and 56% of the identified mosquitoes, respectively. No other member of the complex nor a hybrid *An. gambiae*/*An. arabiensis* was detected. *An. gambiae* was present at all the sampled sites but, although it was more common in T2 than in T1 (Fig. 2A and File S3), no significant difference in abundance means was observed between the two transects (F_1,18_ = 1.72; p = 0.21). Within transects, *An. gambiae* was more common at three sites (Afia, Sare Sidy, and Koar) in T1 and at two sites (Gourel Ndiapalde and Wassadou) in T2. The frequency of *An. gambiae* varied from 26% to 59% in T1 sites and from 36% to 85% in T2 sites (Fig. 2B); there were no significant differences in frequency between villages in either T1 (F_10_,_44_ = 0.26; p = 0.99) or T2 (F_8_,_36_ = 0.38; p = 0.92).

#### Identification of *An. gambiae* molecular forms

**Figure 3:**
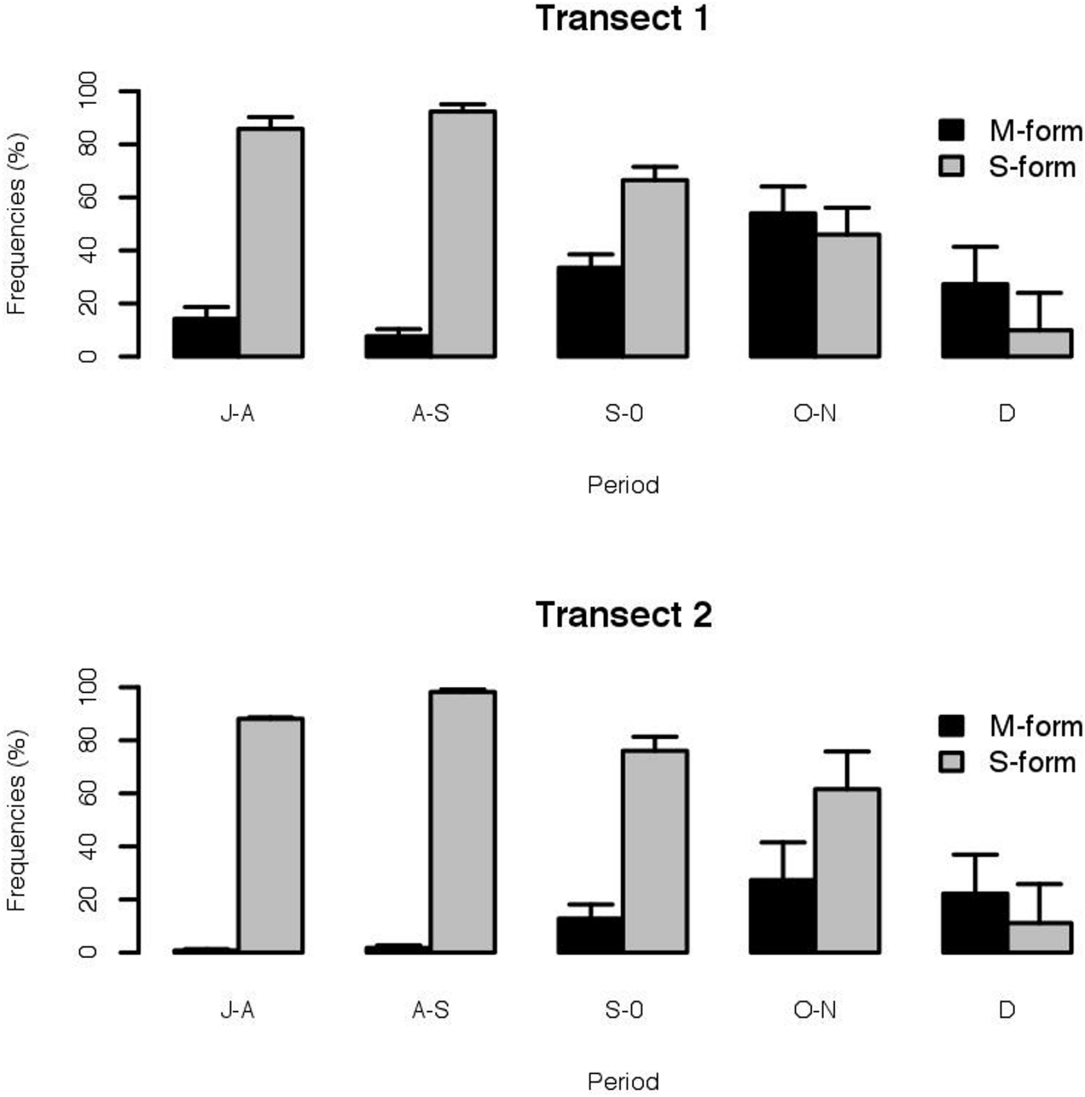
Temporal variations of the mean frequencies of M and S forms in each of the two transects from July to December 2010. Bars represent standard errors. For each period, collections took place from the last week of a given month to the first week of the following month.

Of the 1,225 *An. gambiae* s.s. processed, the S molecular form predominated (82.8%). The two molecular forms of *An. gambiae* s.s. were sympatric at all the study sites in T1 and at eight of the nine sites in T2 (Table 2). No significant difference was observed in the mean frequency of the S form between villages in T1 (F_10_,_44_ = 0.25; p = 0.99) and villages in T2 (F_8_,_36_ = 0.76, p = 0.66). Due to the small sample sizes and the absence of significant differences between study sites, temporal variation was studied at transect level. For both molecular forms, variation in frequency was similar in each transect. For the S form, the highest frequencies were observed at the beginning of the survey, decreasing gradually towards the end of the collections in December. An inverse trend was observed for the M form, which increased in frequency towards the end of the rainy season (Fig. 3 and File S4).

**Table 2:** Frequencies of species within *Anopheles gambiae* complex and of molecular forms of *Anopheles gambiae* s.s. in the two transects.

Transects/Sampling sites	Collected	Sibling species identification			Molecular forms identification					
		N	*An. arabiensis*	*An. gambiae* s.s.	N	M-form	S-form	M/S hybrids		
			*%*	%		%	%	Obs.	Exp.	Fis
Transect 1										
Neteboulou	166	146	60.3	39.7	58	19.0	79.3	1.7	31.8	+0.95
Tourema	231	146	73.3	26.7	39	23.0	74.4	2.6	36.9	+0.93
Gouloumbou	435	300	61.0	39.0	117	16.2	78.6	5.2	30.5	+0.83
Afia	124	94	45.7	54.3	51	11.8	86.2	2.0	22.2	+0.91
Temento Malede	362	156	73.7	26.3	41	19.5	78.0	2.5	32.9	+0.93
Sare Sidy	205	104	46.2	53.8	56	7.1	89.3	3.6	16.3	+0.78
Sankagne	377	174	74.1	25.9	45	26.7	66.6	6.7	42.0	+0.84
Dialiko	126	120	50.8	49.2	59	22.0	78.0	0.0	34.4	+1
Nguene	416	159	61.0	39.0	62	21.0	79.0	0.0	33.1	+1
Koar	310	256	41.0	59.0	151	15.9	83.4	0.7	27.2	+0.98
Saal	324	165	62.4	37.6	62	30.6	69.4	0.0	42.5	+1
Transect 2										
Tamba Soce	50	48	60.4	39.6	19	0.0	94.7	5.3	5.1	–0.03*
Djounkore Mafing	356	213	50.2	49.8	106	2.8	92.5	4.7	9.8	+0.52
Madina Dian	69	66	63.6	36.4	24	8.3	87.5	4.2	18.7	+0.78
Missirah	14	14	64.3	35.7	5	20.0	80.0	0.0	32.0	+1
Barkeyel	180	88	62.5	37.5	33	12.1	87.9	0.0	21.3	+1
Gourel Ndiapalde	100	85	15.3	84.7	72	5.6	93.0	1.4	11.7	+0.88
Bira	74	61	41.0	59.0	36	5.6	94.4	0.0	10.5	+1
Badi	331	226	62.4	37.6	85	29.4	70.6	0.0	41.5	+1
Wassadou	348	162	35.8	64.2	104	6.7	92.3	1.0	13.4	+0.93

% = Percentage, Obs. = observed, Exp. = expected, *Fis* = inbreeding coefficient calculated according to Weir and Cockerham [37], *Fis*<0 indicate an excess of heterozygotes, *Fis*>0 denote heterozygotes deficiency, *no significant deviation from Hardy–Weinberg expectations (P>0.05).

### Spatial analysis of M/S hybrid distribution

**Figure 4:**
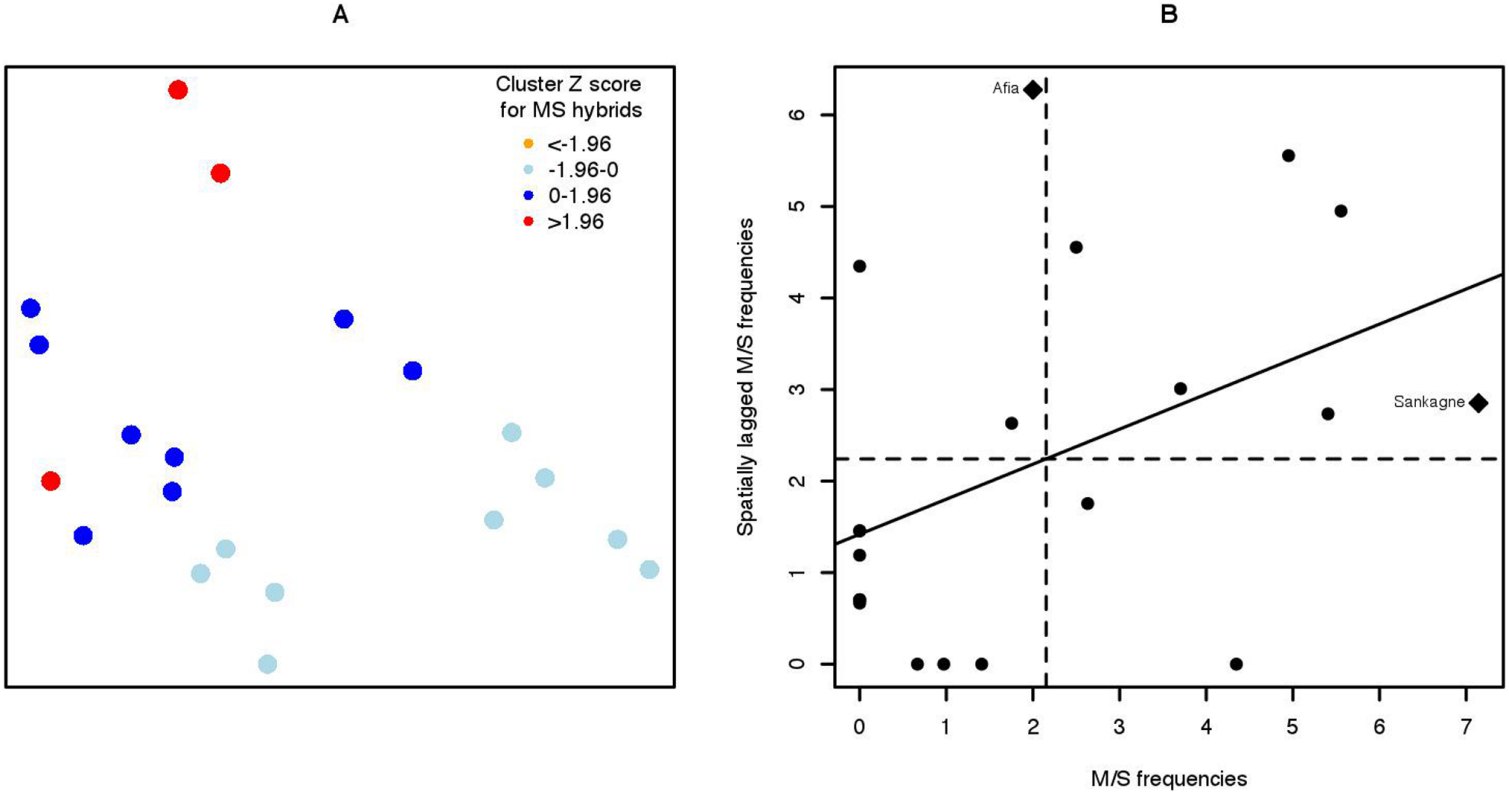
Spatial distribution of M/S hybrids frequencies. Spatial clustering trends (A). Significance of clustering was analysed using the Getis-Ord Gi* statistic [19]. (Z scores>0 indicates a clustering trend of high MS hybrids frequencies and Z scores<0 indicates a clustering trend of low MS hybrids frequencies. Significant Z scores (p<0.05) are in red. Moran scatter plots at site level (B). The names of the sites with large contributions to autocorrelation are displayed.

Overall, the frequency of M/S hybrids was estimated at 2.16% in T1 (range: 0–6.7%) and 1.86% in T2 (range: 0–5.3%). The highest frequencies were observed in the north-western parts of both transects (Fig. 4A). Analysis of M/S hybrid frequencies showed significant deviations from the Hardy-Weinberg equilibrium in all samples, except for that collected in the village of Tamba Soce (Table 2).

Spatial analysis indicated positive spatial autocorrelation or clustering for M/S hybrids (Moran’s *I* value = 0.38; Z score = 0.002; p = 0.03). The resultant Z scores for the Getis-Ord Gi* hot spot analysis (using inverse-distance weighting) indicated the presence of spatial clusters of M/S hybrid frequencies. The clustering trends are shown in Fig. 4A. For T1, seven locations, predominantly in the north-eastern part, had positive Z scores (ranging from 0.03–2.22), while the remaining four locations, predominately in the south-western part, had negative Z scores (ranging from –1.94–1.04). For T2, two locations in the north-eastern part with high frequencies of M/S hybrids clustered significantly (Z scores>1.96), whereas five locations in the south-western part had negative Z scores (ranging from –1.76–1.09).

On the Moran scatter plot of M/S hybrid frequencies, sampling units at the site level were distributed in the four quadrants of the scatter plot, and all four types of association between spatial units (High-High, High-Low, Low-High, Low-Low) were represented (Fig. 4B). The dominant types of association were represented by Low-Low (cold spots) and High-High (hot spots) with four sites for both types in T1 and two cold spots and five hot spots in T2. Two sampling sites (both located in T1) with high influence on the global autocorrelation were identified (Afia in the Low-High and Sankagne in the High-High quadrants). In both transects, the mean M/S frequencies differed significantly between High-High and Low-Low clusters (Table 3).

**Table 3:** Characteristics of clusters classes.

Transects	Characteristics	Clusters classes			
	of clusters	High-High	High-Low	Low-High	Low-Low
Transect 1	MS range	2.5–7.14	2.63	1.75–2.00	0–0.67
	Mean MS±SE	4.69±1.01^a^	2.63^a, b^	1.88±0.12^a, b^	0.17±0.17^b^
	Sampling sites	4	1	2	4
Transect 2	MS range	4.95–5.55	4.35	0	0–1.41
	Mean MS±SE	5.25±0.30^a^	4.35^a^	0^b^	0.47–0.30^b^
	Sampling sites	2	1	1	5

MS = MS hybrids frequencies.

For the different clusters, means with different letters are significantly different (p<0.05).

## Discussion

These results provide a spatial and temporal picture of the distribution of species and molecular forms of *An. gambiae* in an inland area of eastern Senegal, as well as the variation in the frequency of M/S forms. The absence of *An. melas* from our samples is most likely due to our sampling sites being situated far from coastal regions. Unlike previous entomological studies conducted in the southern zone of Senegal [6], [20], [21], we found that *An. arabiensis* was more common than *An. gambiae*. It is possible, given the zoo-anthropophilic and exophilic behaviour of this species, that collection methodology biased the sampling; however, as we used the same collection methods as previous studies, the difference in our results may result from an increase in the use of insecticides in this zone to control endophagic vectors since the earlier studies were conducted. Our current observations support recent results indicating that *An. gambiae* s.s. is the prevailing species in coastal regions of The Gambia and surrounding areas in Senegal, whilst its sibling species, *An. arabiensis*, is more common in inland areas [10], [11]. Similar observations were also made by Onyabe and Conn [22] and Kristan et al. [23], who found *An. arabiensis* more abundant in the Guinean region in the south of Nigeria. These results indicate that *An. arabiensis* is extending its range southwards, from drier savannah into humid forest habitats. Such a process could result from drought and/or human activity (deforestation and urbanization), as suggested by Lindsay and Martens [24]. We note, however, that in our study *An. arabiensis* was more abundant in inland areas along the same latitudinal line and, thus, the causes of the extended range of *An. arabiensis* may result from other as yet unidentified factors. These might include the types of breeding sites provided by marshes and rice fields, which are more prevalent habitats in areas with the highest densities of *An. gambiae* [10], [25].

The S molecular form was prevalent in almost all our study sites throughout the study period. This is consistent with previous observations that the S form is more common than the M form in eastern Senegal [6], [11]. In West Africa, the two forms are frequently found in sympatry from the Sudan–Guinea savannahs, below 13° N, to the more humid and forested areas, above 4° S, including Senegal [6].

Several studies suggest that the nature of larval habitats is the principal ecological factor responsible for divergence between molecular forms within the *An. gambiae* complex [26],[27],[28],[29]. In rural sites situated within freshwater floodplains, the presence of numerous rain-dependent breeding habitats in the form of temporary water bodies such as puddles, tyre tracks and animal footprints may produce conditions more favourable to the S form. This idea is supported by the more frequent presence of the M form in areas with a greater number of permanent breeding sites [30] and by studies showing its increased abundance in arid areas and during the dry season, when only permanent oviposition sites are available [28]. We also observed higher frequencies of the M form towards the end of the rainy season (November–December), when permanent breeding sites become more prevalent; however, a similar ecological division is absent from Central Africa, emphasizing the difficulty in generalizing putative phenotypic differences from studies conducted in a single area [27]. Local adaptation within forms may be common, as extensive genetic studies highlighted genetic subdivision within the M form [31], [32], [33], [34], and such differences may explain the observed patterns of distribution.

We identified and genotyped 1,207 *An. gambiae s.s.* and found M/S hybrids were present at frequencies of up to 7%. This is a relatively high rate of hybridization in comparison with the 1% heterogamous insemination recorded in several west African populations [2], [6], [35], but low when compared with populations from “far-west” Africa [10], [11], [12]. Therefore, the breakdown in reproductive isolation observed in coastal areas of The Gambia, Senegal and Guinea-Bissau is not observed in this inland zone. However, in our study, sites with relatively high hybridization rates clustered near urban settings in the north-western part of both transects; thus, the high hybridization rates could result from anthropogenic impacts. This is consistent with the results of Kamdem et al. [36] who showed in a central African rainforest the presence of M/S hybrids only in a peri-urban zone of sympatry and that the pattern of occurrence of molecular forms was spatially structured, and that the frequency of each form within a locality was not independent of the frequencies in surrounding sites. In our sampling plan, distances between collection sites were set at 5–10 km, and so non-independence of samples may account in part for the observed patterns of clustering in these areas.

We found substantial reproductive isolation between the M and S forms in many sites, adding further support to the proposition that a speciation process is currently underway in *An. gambiae*. However, the results also indicate that the breakdown of this process seen in coastal areas [12] is absent from inland sites. The M and S molecular forms in the present study correspond to *An. coluzzii* Coetzee & Wilkerson sp.n. and *An. gambiae* Giles, respectively, as described in a recent reclassification of the *An. gambiae* species complex [8]. The application of whole-genome studies should further provide more information about the extent of the genetic isolation. From applications perspective, future scaling up of insect control strategies based on insecticide-impregnated materials and indoor residual spraying will need to take into account the mounting evidence that molecular forms differ in their utilization of breeding sites and their response to environmental change. Therefore, further detailed study of these behavioural and ecological characteristics will allow the development and implementation of better control strategies for malaria.

## Supporting Information

File S1 **Table, Detailed characteristics of the studied villages.**
(PDF)

File S2 Text, Estimation of Global Moran’s I statistic and Local Gi*(d) statistic. Table, Z scores for each of the 20 sites.
(PDF)

File S3 **Figures, Temporal variations of the frequencies of** ***An. arabiensis*** **and** ***An. gambiae*** **in each of the two transects from July to december 2010.** Table, Comparison of the mean frequencies of *An. gambiae* and *An. arabiensis* between the two transects.
(PDF)

File S4 **Figures.** Temporal variations of the M/S hybrids frequencies in each of the two transects. Table. Comparison of mean M/S hybrids frequencies between the two transects.
(PDF)
