# Supplementary material for: Reproductive Isolation among Sympatric Molecular Forms of An. gambiae from Inland Areas of South-Eastern Senegal
Source: PLoS One. 2014 Aug 6;9(8):e104622. doi: 10.1371/journal.pone.0104622 (PMC4123975; doi:10.1371/journal.pone.0104622)
Supplement: File S1 — Table, Detailed characteristics of the studied villages. (PDF) [file pone.0104622.s001.pdf]

Supporting Information S1

Table: Detailed characteristics of the studied villages

Transects	Sampling Sites	Latitude N	Longitude W	Area type	Main human activities	Distance to nearest water points	Habitats*	Use of pesticide	Presence of LLINs	No. houses	Livestock
Transect 1	Neteboulou	13°34'70.0"	13°47'08.2"	Semi-urban	Agro pastoral	Neteboulou river<1km	corrugated iron	Yes	Yes	111	Ovine, capri[illegible] poultry
	Tourema	13°32'75.3"	13°46'47.1"	Rural	Agro pastoral	Gouloumbou river (1km)	corrugated iron, thatched roofs	Yes	Yes	14	Bovine, poul[illegible]
	Gouloumbou	13°27'93.1"	13°42'59.1"	Semi-urban	Agro pastoral & trade	Gouloumbou river (5m)	corrugated iron	Yes	Yes	15	ovine, capri[illegible] volaille
	Afia	13°25'68.0"	13°46'18.3"	Semi-urban	Cash crop (Banana)	Gouloumbou river <2km	corrugated iron, thatched roofs	Yes	Yes	503	ovine, capri[illegible] volaille
	Temento Malede	13°26'83.3"	13°41'11.9"	Rural	Agro pastoral	Niaoule river à 500m	thatched roofs	Yes	Yes	11	bovin et vol[illegible]
	Sare Sidy	13°25'35.0"	13°40'77.1"	Rural	Agro pastoral	Niaoule river <3km	corrugated iron, thatched roofs	Yes	Yes	24	bovin et vol[illegible]
	Sankagne	13°23'17.0"	13°44'58.0"	Semi-urban	Cash crop (Banana)	Gouloumbou <2km	corrugated iron	Yes	Yes	525	ovine, capri[illegible] volaille
	Dialiko	13°21'95.1"	13°38'64.5"	Rural	Agro pastoral	Gouloumbou <3km	thatched roofs, corrugated iron	Yes	Yes	122	bovine, ovin[illegible] caprine, pou[illegible]
	Nguene	13°21'18.2"	13°40'07.1"	Rural	Cash crop (Banana)	Gouloumbou <1km	thatched roofs	Yes	Yes	55	bovine, ovin[illegible] caprine, pou[illegible]
	Koar	13°20'19.16"	13°37'02.86"	Rural	Cash crop (Banana)	Gouloumbou <2km	thatched roofs	Yes	Yes	145	bovine, ovin[illegible] caprine, pou[illegible]
	Saal	13°16'33.9"	13°36'81.0"	Rural	Cash crop (Banana)	Gouloumbou <2km	thatched roofs	Yes	Yes	93	bovine, ovin[illegible] caprine, pou[illegible]
Transect 2	Tamba Soce	13°46'35.0"	13°40'62.8"	Semi-urban	Agro pastoral	Rainy temporary pools	corrugated iron, thatched roofs	Yes	Yes	50	ovine, capri[illegible] volaille
	Djounkore Mafing	13°41'73.5"	13°38'77.4"	Rural	Agro pastoral	Rainy temporary pools	thatched roofs, corrugated iron	Yes	Yes	12	ovine, capri[illegible] volaille
	Madina Dian	13°33'96.4"	13°33'71.6"	Rural	Agro pastoral & trade	Rainy temporary pools	thatched roofs, corrugated iron	Yes	Yes	44	ovine, capri[illegible] volaille
	Missirah	13°31'53.8"	13°30'81.2"	Urban	Agro pastoral & trade	Rainy temporary pools	corrugated iron, thatched	Yes	Yes	800	ovine, capri[illegible] volaille

						roofs				
Barkeyel	13°28'40.47"	13°27'15.62"	Rural	Agro pastoral	Rainy temporary pools	thatched roofs, corrugated iron	Yes	Yes	35	bovine, ovin[illegible] caprine, poul[illegible]
Gourel Ndiapalde	13°26'17.8"	13°25'53.4"	Rural	Agro pastoral & trade	Rainy temporary pools	thatched roofs, corrugated iron	Yes	Yes	35	ovine, capri[illegible] poultry
Bira	13°24'05.91"	13°28'00.04"	Rural	Agro pastoral	Rainy temporary pools	thatched roofs, corrugated iron	Yes	Yes	131	ovine, capri[illegible] poultry
Badi	13°22'65.3"	13°22'53.3"	Rural	Agro pastoral & trade	Nieriko river <2km	thatched roofs, corrugated iron	Yes	Yes	53	ovine, capri[illegible] poultry
Wassadou	13°21'30.8"	13°20'94.1"	Rural	Agro pastoral & trade	Nieriko river (500m)	thatched roofs, corrugated iron	Yes	Yes	214	ovine, capri[illegible] poultry

*the first is more abundant
