# Supplementary material for: Reproductive Isolation among Sympatric Molecular Forms of An. gambiae from Inland Areas of South-Eastern Senegal
Source: PLoS One. 2014 Aug 6;9(8):e104622. doi: 10.1371/journal.pone.0104622 (PMC4123975; doi:10.1371/journal.pone.0104622)
Supplement: File S2 — Text, Estimation of Global Moran’s I statistic and Local Gi*(d) statistic. Table, Z scores for each of the 20 sites. (PDF) [file pone.0104622.s002.pdf]

SUPPORTING INFORMATION S2

Text: Estimation of Global Moran's I statistic and Local Gi*(d) statistic

- Global Moran's I statistic

The global spatial autocorrelation statistical method was used to measure the correlation among neighboring sites, to find the patterns and the levels of spatial clustering. The Moran's I statistic is calculated by the following formula:

$$I = \frac{N}{S_O} \sum_i \sum_j w_{ij} \frac{(x_i - u)(x_j - u)}{\sum_i (x_i - u)^2}, \qquad (1)$$

where *N* is the number of sites; *wij* is the element in the spatial weight matrix corresponding to the observation pair *i*, *j*; and *xi* and *xj* are observations for areas *i* and *j* with mean *u* and

$$S_O = \sum_i \sum_j w_{ij} \qquad (2)$$

The first step in the spatial autocorrelation analysis is to construct a spatial weight matrix that contains information about the neighborhood structure for each site. Adjacency is defined as immediately neighboring sites, inclusive of the site itself. Non-neighboring sites are given a weight of zero.

- Local Gi*(d) statistic

The local *Gi *(d)* statistic (local G-statistic) is used to test the statistical significance of local clusters of M/S hybrids frequencies, and to determine the spatial extent of these clusters. The local G-statistic is useful for identifying individual members of local clusters by determining the spatial dependence and relative magnitude between a site and neighboring sites. The local G-statistic can be written as follows:

$$G_i^*(d) = \frac{\sum_j w_{ij}(d) x_j - W_i \bar{x}}{s\sqrt{\frac{\left(nS_{1i} - W_i^2\right)}{(n-1)}}}, \text{ for all } j \qquad (3)$$

where *x* is a measure of the M/S hybrids frequencies within a given polygon (i.e., each site); *wij* is a spatial weight that defines neighboring sites *j* to *i*; *Wi* is the sum of the weights *wij*,

$$\bar{x} = \frac{1}{n} \sum_j x_j \quad S_{1i} = \sum_j w_{ij}^2, \quad s^2 = \frac{1}{n} \sum_j x_j^2 - \bar{x}^2 .$$

Developing the spatial weights *wij* is the first step to calculating *Gi*(d)*. The spatial weight matrix includes *wij* = 1. Non-neighboring sites are given a weight of zero.

The local G-statistic includes the value in the calculation at *i*. Assuming that *Gi*(d)* is approximately normally distributed, the output of *Gi*(d)* can be calculated as a standard normal variant with an associated probability from the z-score distribution. Statistically significant (at a level of 0.05) clusters of sites with high M/S frequencies were identified with Z scores >1.96. Clustered sites with low M/S frequencies were identified with Z scores <-1.96.

Sites	Z scores

Neteboulou	0,02680303
Tourema	0,02680303
Gouloumbou	1,22336807
Afia	2,21740575
Temento Malede	1,41206487
Sare Sidy	1,24698827
Sankagne	1,75113205
Dialiko	-1,0364073
Nguene	-1,0364073
Koar	-1,9423252
Saal	-1,1861733
Tamba Soce	2,02274922
Djounkore Mafing	2,02274922
Madina Dian	0,0143648
Missirah	0,0143648
Barkeyel	-1,3829601
Gourel Ndiapalde	-1,7608864
Bira	-1,3829601
Badi	-1,1169364
Wassadou	-1,0869621

Table: Z scores for each of the 20 sites
(Z score>0 indicates a clustering trend of high MS hybrids frequencies and Z score<0 indicates a clustering trend of low MS hybrids frequencies.

References

Moran PAP (1948) The interpretation of statistical maps. J R Stat Soc Series B Stat Methodol 10:243–251.

Getis A, Ord JK (1992) The analysis of spatial association by use of distance statistics. Geogr Anal 24:189–206.
