# Supplementary material for: Reproductive Isolation among Sympatric Molecular Forms of An. gambiae from Inland Areas of South-Eastern Senegal
Source: PLoS One. 2014 Aug 6;9(8):e104622. doi: 10.1371/journal.pone.0104622 (PMC4123975; doi:10.1371/journal.pone.0104622)
Supplement: File S3 — Figures, Temporal variations of the frequencies of An. arabiensis and An. gambiae in each of the two transects from July to december 2010. Table, Comparison of the mean frequencies of An. gambiae and An. arabiensis between the two transects. (PDF) [file pone.0104622.s003.pdf]

SUPPORTING INFORMATION S3

Figures. Temporal variations of the frequencies of *An. arabiensis* and *An. gambiae* in each of the two transects from July to december 2010.

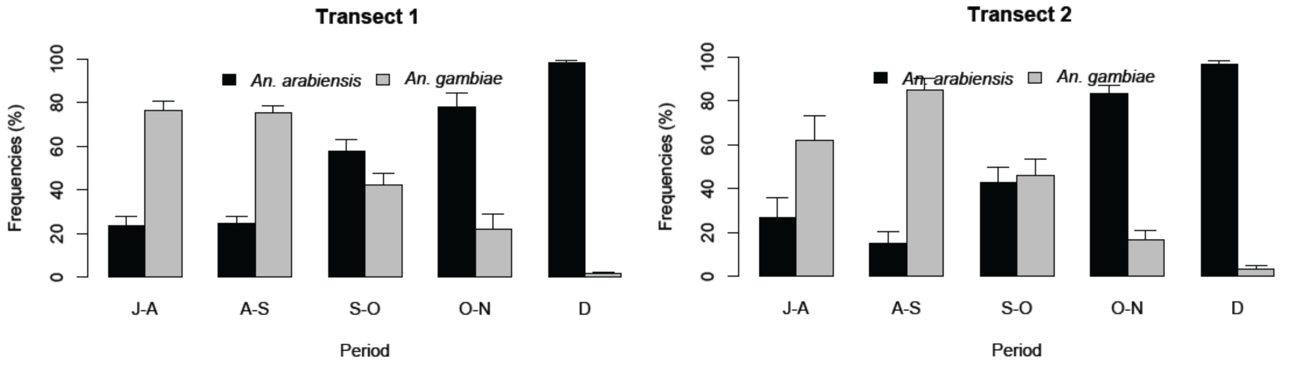

A similar trend is observed for the two transects when considering the tow species. The highest frequencies of *An. arabiensis* are observed at the end of the rainy season whereas for *An. gambiae* the highest frequencies are observed at the beginning of the rainy season. These variations were significantly different (see table below).

Table: Comparison of the mean frequencies of *An. gambiae* and *An. arabiensis* between the two transects

Transects/Period			J-A	A-S	S-O	O-N	D	F	p
Transect 1	*An. arabiensis*	mean	23.72	24.61	57.62	77.83	98.50	53.01	<0.001
		±se	4.25	3.18	5.32	6.66	0.90		
	An. gambiae	mean	76.28	75.39	42.38	22.17	1.50		
		±se	4.25	3.18	5.32	6.66	0.90		
Transect 2	*An. arabiensis*	mean	26.92	15.04	42.85	83.24	96.77	35.18	<0.001
		±se	9.06	5.37	6.97	4.10	1.80		
	An. gambiae	mean	61.97	84.96	46.04	16.76	3.23		
		±se	11.43	5.37	7.28	4.10	1.80		
