# Supplementary material for: Reproductive Isolation among Sympatric Molecular Forms of An. gambiae from Inland Areas of South-Eastern Senegal
Source: PLoS One. 2014 Aug 6;9(8):e104622. doi: 10.1371/journal.pone.0104622 (PMC4123975; doi:10.1371/journal.pone.0104622)
Supplement: File S4 — Figures. Temporal variations of the M/S hybrids frequencies in each of the two transects. Table. Comparison of mean M/S hybrids frequencies between the two transects. (PDF) [file pone.0104622.s004.pdf]

SUPPORTING INFORMATION S4

Figures. Temporal variations of the MS hybrids frequencies in each of the two transects

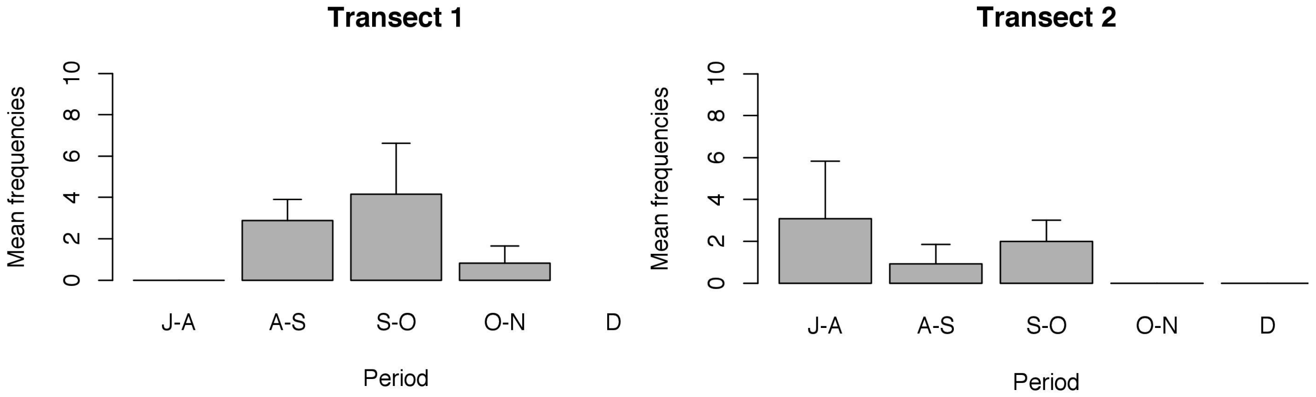

In the transect 1, the MS hybrids frequencies increased gradually from the beginning of the survey to reach a peak in September-October whereas in transect 2, the highest frequencies was observed at the beginning of the survey. While in transect 1, no hybrid was observed in December, in transect 2, no hybrid was observed both in October-November and December.
In both transects, no significant difference was observed between the different months of collection (see Table below).

Table: Comparison of mean MS hybrids frequencies between the two transects

Transects/Period		J-A	A-S	S-O	O-N	D	F	p
Transect 1	mean	0	2.88	4.14	0.83	-	2.12	0.09
	se	0	1.02	2.48	0.83	-		
Transect 2	mean	3.08	0.92	1.99	0	0	0.93	0.45
	se	2.76	0.92	1.01	0	0		
